# Supplementary material for: First Report on Leptospira Species Isolated from Patients in Slovenia
Source: Microorganisms. 2023 Nov 9;11(11):2739. doi: 10.3390/microorganisms11112739 (PMC10672770; doi:10.3390/microorganisms11112739)
Supplement: Supplementary file 1 [file microorganisms-11-02739-s001.zip › microorganisms-2632361-supplementary.pdf]

Table S1. Rabbit immune sera used for *Leptospira* serotyping.

No.	Name of immune serum[1]
1	Australis, Australis (Ballico)
2	Autumnalis, Autumnalis (Akiyami A)
3	Bataviae, Bataviae, (Van Tienen)
4	Ballum, Castellonis
5	Canicola, Canicola
6	Cynopteri, Cynopteri (3522C)
7	Grippotyphosa, Grippotyphosa
8	Icterohaemorrhagiae, Copenhageni
9	Javanica, Javanica
10	Panama, Panama (CZ214K)
11	Pomona, Pomona
12	Pyrogenes, Pyrogenes (Salinem)
13	Semaranga, Patoc
14	Sejroe, Sejroe (M84)
15	Tarassovi, Tarassovi (Mitis Johnson)

[1] Immune sera obtained from the Royal Tropical Institut, Amsterdam.

Table S2: Species identification of *Leptospira* strains isolated from patients in Slovenia and reference strains using PCR-Tm analysis.

Strain	Melting temperature (Tm)			Average Tm	Identification
	Tm 1	Tm 2	Tm 3		
SS 166/02	83.72	83.68	83.54	83.61	*L. interrogans*
ČP 226/02	83.75	83.77	83.44	83.65	*L. interrogans*
OD 8720/11	83.75	83.68	83.68	83.70	*L. interrogans*
KB 185/02	84.77	84.81	84.83	84.65	*L. kirschneri*
VV 206/02	84.81	84.86	84.38	84.82	*L. kirschneri*
FF 291/02	84.80	84.85	84.21	84.62	*L. kirschneri*
PZ 9474/06	84.80	84.87	84.66	84.78	*L. kirschneri*
SS 8049/14	84.45	84.72	84.79	84.65	*L. kirschneri*
MJ 112721/08	86.72	86.65	86.51	86.62	*L. borgpetersenii*
DDA 10944/10	86.68	86.62	87.31	86.87	*L. borgpetersenii*
Australis Australis Ballico	84.09	83.68	84.47	84.08	*L. interrogans*
Autumnalis Autumnalis Akiyami A	83.77	83.49	83.42	83.56	*L. interrogans*
Bataviae Bataviae Van Tienen	83.73	83.95	83.85	83.84	*L. interrogans*
Ballum Castellanis Castellon 3	86.62	86.11	86.51	86.41	*L. borgpetersenii*
Canicola Canicola Hond Utrecht Iv	83.80	83.95	83.90	83.88	*L. interrogans*
Cynopteri Cynopteri 3522c	84.67	84.42	84.86	84.65	*L. kirschneri*
Grippothyphosa Grippotyphosa Moskvav	84.63	84.83	84.59	84.68	*L. kirschneri*
Icterohaemorrhagiae Copenhageni Wijnberg	83.06	83.78	83.52	83.45	*L. interrogans*
Javanica Javanica Veldart Batavia 46	86.6	86.38	86.45	86.48	*L. borgpetersenii*
Panama Panama Cz 214 K	84.99	84.88	84.57	84.81	*L. noguchii*
Pomona Pomona Pomona	83.83	84.1	83.97	83.97	*L. interrogans*
Pyrogenes Pyrogenes Salinem	83.96	84.17	84.04	84.06	*L. interrogans*
Semaranga Patoc Patoc 1	–	–	–	–	not possible[1]
Sejroe Sejroe M84	86.20	86.60	86.32	86.37	*L. borgpetersenii*
Tarassovi Tarassovi Mitis Johnson	86.66	86.47	86.63	86.59	*L. borgpetersenii*

[1]Saprophytic *Leptospira* could not be amplified using primers for pathogenic *Leptospira*.

Table S3: Serotyping clinical and reference *Leptospira* strains using immune sera

Strain	Serogroup identification	Titer
SS 166/02	Icterohaemorrhagiae	1 : 800
ČP 226/02	Icterohaemorrhagiae	1 : 1600
OD 8720/11	Icterohaemorrhagiae	1 : 3200
KB 185/02	Grippotyphosa	1 : 1600
VV 206/02	Grippotyphosa	1 : 6400
FF 291/02	Grippotyphosa	1 : 6400
PZ 9474/06	Grippotyphosa	1 : 12 800
SS 8049/14	Grippotyphosa	1 : 1600
MJ 112721/08	Sejroe	1 : 400
DDA 10944/10[1]	Bataviae	1 : 800
LA 9525/17	Sejroe	1:51 000
PM 155/20	Sejroe	1:800
Australis Australis Ballico	Australis	1 : 12 800
Autumnalis Autumnalis Akiyami A	Autumnalis	1 : 25 600
Bataviae Bataviae Van Tienen	Bataviae	1 : 800
Ballum Castellanis Castellon 3	Ballum	1 : 12 800
Canicola Canicola Hond Utrecht Iv	Canicola	1 : 25 600
Cynopteri Cynopteri 3522c	Cynopteri	1 : 800
Grippothyphosa Grippotyphosa Moskva V	Grippotyphosa	1 : 3200
Icterohaemorrhagiae Copenhageni Wijnberg	Icterohaemorrhagiae	1 : 1600
Javanica Javanica Veldart Batavia 46	Javanica	1 : 12 800
Panama Panama Cz 214 K	Panama	1 : 12 800
Pomona Pomona Pomona	Pomona	1 : 12 800
Pyrogenes Pyrogenes Salinem	Pyrogenes	1 : 800
Semaranga Patoc Patoc 1	Semaranga	1 : 12 800
Sejroe Sejroe M84	Sejroe	1 : 12 800
Tarassovi Tarassovi Mitis Johnson	Tarassovi	1 : 6400

[1]Isolate taken from a South American patient who was infected with *Leptospira* while travelling through Asia. The patient became ill and was hospitalized in Slovenia.

Table S4: Allele numbers and sequence types (ST) of clinical and reference *Leptospira* strains

Strain	Allele number							ST	Species
	tpiA	sucA	pntA	pfkB	mreA	glmU	caiB		
SS 166/02	2	2	1	10	4	1	8	17	*L. interrogans*
ČP 226/02	2	2	1	10	4	1	8	17	*L. interrogans*
OD 8720/11	2	2	1	10	4	1	8	17	*L. interrogans*
KB 185/02	22	13	20	31	18	19	23	110	*L. kirschneri*
VV 206/02	22	13	20	31	18	19	23	110	*L. kirschneri*
FF 291/02	22	13	20	31	18	19	23	110	*L. kirschneri*
PZ 9474/06	22	13	20	31	18	19	23	110	*L. kirschneri*
SS 8049/14	22	13	20	31	18	19	23	110	*L. kirschneri*
MJ 12721/08	2	2	28	9	18 or 27	24	28	Not specified	–[1]
DDA 10944/10	2	47	55	4 or 20	44	19	43	Not specified	–[1]
Australis Australis Ballico	2	2	13	13	2	6	6	51	*L. interrogans*
Autumnalis Autumnalis Akiyami A	3	3	12	10	6	2	1	27	*L. interrogans*
Bataviae Bataviae Van Tienen	2	2	8	9	7	6	5	50	*L. interrogans*
Ballum Castellanis Castellon 3	36	30	32	67	26	24	12	149	*L. borgpetersenii*
Canicola Canicola Hond Utrecht Iv	3	3	3	4	5	3	5	37	*L. interrogans*
Cynopteri Cynopteri 3522c	22	15	22	33	18	17	10	70	*L. kirschneri*
Grippothyphosa Grippotyphosa Moskva V	22	13	20	31	18	19	23	110	*L. kirschneri*
Icterohaemorrhagiae Copenhageni Wijnberg	2	2	1	10	4	1	8	17	*L. interrogans*
Javanica Javanica Veldart Batavia 46	34	30	27	67	27	24	11	143	*L. borgpetersenii*
Panama Panama Cz 214 K	46	41	47	50	35	39	36	171	*L. noguchii*
Pomona Pomona Pomona	3	3	3	4	5	3	16	140	*L. interrogans*
Pyrogenes Pyrogenes Salinem	5	18	1	12	2	5	1	88	*L. interrogans*
Semaranga Patoc Patoc 1	/	/	/	/	/	/	/	/	–[1]
Sejroe Sejroe M84	/	/	/	/	/	/	/	/	–[1]
Tarassovi Tarassovi Mitis Johnson	/	/	/	/	/	/	/	/	–[1]

[1]Identification was not possible because the strain was not found in the applied MLST scheme.

Table S5: Identification of clinical and reference *Leptospira* strains using matrix-assisted laser desorption/ionisation time of flight mass spectrometry (MALDI-TOF) mass spectra.

Species	Strain	MALDI-TOF species identification	MALDI-TOF score value
	SS 166/02	*Leptospira* sp. (*L. interrogans*)[3]	1.947[3]
	ČP 226/02	*L. interrogans*[2]	2.065[2]
	OD 8720/11	*L. interrogans*[1]	2.503[1]
	FF 291/02	*Leptospira* sp. (*L. interrogans*)[3]	1.739[3]
	SS 8049/14	*Leptospira* sp. (*L. kirschneri*)[3]	1.999[3]
	MJ 112721/08	*L. borgpetersenii*[2]	2.239[2]
	DDA 10944/10	*Leptospira* sp. (*L. santarosai*)[3]	1.992[3]
L. interrogans	Australis Australis Ballico	*L. interrogans*	2.749
L. interrogans	Autumnalis Autumnalis Akiyami A	*L. interrogans*	2.537
L. interrogans	Bataviae Bataviae Van Tienen	*L. interrogans*	2.722
L. borgpetersenii	Ballum Castellanis Castellon 3	*L. borgpetersenii*	2.630
L. interrogans	Canicola Canicola Hond Utrecht Iv	*L. kirschneri*	2.670
		L. interrogans	2 609
L. kirschneri	Cynopteri Cynopteri 3522C	*L. interrogans*	2.617
		L. kirschneri	2 603
L. kirschneri	Grippothyphosa Grippotyphosa Moskvav	*L. kirschneri*	2.752
L. interrogans	Icterohaemorrhagiae Copenhageni Wijnberg	*L. interrogans*	2.833
L. noguchii	Panama Panama Cz 214 K	*L. noguchii*	2.411
L. borgpetersenii	Sejroe Sejroe M84	*L. borgpetersenii*	2.623
L. borgpetersenii	Tarassovi Tarassovi Mitis Johnson	*L. borgpetersenii*	2.679
L. interrogans	Pomona Pomona Pomona	*L. interrogans*	2.765
L. interrogans	Pyrogenes Pyrogenes Salinem	*L. interrogans*	2.665
L. biflexa	Semaranga Patoc Patoc 1	*L. biflexa*	2.683

[1]Highly probable species identification.

[2]Secure genus identification, probable species identification.
[3]Probable genus identification.

Table S6: Comparison of the phenotyping and genotyping results for the reference *Leptospira* strains using five different typing methods

Strain	Serotyping	*NotI*-RFLP	PCR-Tm	MLST 1	MALDI-TOF
Australis Australis Ballico	Australis	Australis	*L. interrogans*	*L. interrogans* Australis	*L. interrogans*
Autumnalis Autumnalis Akiyami A	Autumnalis	Autumnalis	*L. interrogans*	*L. interrogans*	*L. interrogans*
Bataviae Bataviae Van Tienen	Bataviae	Bataviae	*L. interrogans*	*L. interrogans*	*L. interrogans*
Ballum Castellanis Castellon 3	Ballum	Not possible[1]	*L. borgpetersenii*	*L. borgpetersenii* Ballum	*L. borgpetersenii*
Canicola Canicola Hond Utrecht Iv	Canicola	Canicola	*L. interrogans*	*L. interrogans*	*L. kirschneri* *L. interrogans*
Cynopteri Cynopteri 3522c	Cynopteri	Cynopteri	*L. kirschneri*	*L. kirschneri*	*L. interrogans* *L. kirschneri*
Grippothyphosa Grippotyphosa Moskva V	Grippotyphosa	Grippotyphosa	*L. kirschneri*	*L. kirschneri* Grippotyphosa	*L. kirschneri*
Icterohaemorrhagiae Copenhageni Wijnberg	Icterohaemorrhagiae	Icterohaemorrhagiae	*L. interrogans*	*L. interrogans* Icterohaemorrhagiae	*L. interrogans*
Javanica Javanica Veldart Batavia 46	Javanica	Javanica	*L. borgpetersenii*	*L. borgpetersenii* Javanica	Not possible[4]
Panama Panama Cz 214 K	Panama	Panama	*L. noguchii*	*L. noguchii* Panama Panama	*L. noguchii*
Pomona Pomona Pomona	Pomona	Pomona	*L. interrogans*	*L. interrogans*	*L. interrogans*
Pyrogenes Pyrogenes Salinem	Pyrogenes	Pyrogenes	*L. interrogans*	*L. interrogans* Pyrogenes Pyrogenes	*L. interrogans*
Semaranga Patoc Patoc 1	Semaranga	Semaranga	Not possible[2]	Not possible[3]	*L. biflexa*
Sejroe Sejroe M84	Sejroe	Sejroe	*L. borgpetersenii*	Not possible[3]	*L. borgpetersenii*
Tarassovi Tarassovi Mitis Johnson	Tarassovi	Not possible[1]	*L. borgpetersenii*	Not possible[3]	*L. borgpetersenii*

MALDI-TOF = matrix-assisted laser desorption/ionisation time-of-flight mass spectrometry.

MLST 1 = multilocus sequence typing scheme 1 according to Boonsilp et al.

PCR-Tm = melting temperature PCR.

NotI-RFLP = *NotI*-restriction fragment length polymorphism

[1] *NotI*-RFLP not precisely defined.
[2] Saprophytic *Leptospira* could not be amplified using primers for pathogenic *Leptospira*.
[3] Identification not possible because strain was not found in the applied MLST scheme.
[4] Identification not possible because of low concentration of *Leptospira* in sample.

Table S7: Whole genome sequencing quality metrix for *Leptospira* strains isolated from Slovenian patients

Sample	species id	KmerFinder	total query coverage	total template coverage	contig count	N50	GC content	approximated genome size (Mbases)
SS 166/02	*L. interrogans*	copenhageni str. FDAARGOS	92,68	99,78	1104	73303	35.1	4.7
ČP 226/02	*L. interrogans*	copenhageni str. FDAARGOS	92,68	99,78	1102	79692	35.1	4.7
OD 8720/11	*L. interrogans*	copenhageni str. FDAARGOS_203	92,68	99,77	792	67739	35.1	4.7
FF 291/02	*L. kirschnerii*	strain FMAS_PN5	60,51	63,55	696	80507	35.9	4.4
SS 8049/14	*L. kirschnerii*	strain FMAS_PN5	60,49	63,56	1062	60528	36.3	4.5
MJ 11721/08	*L. borgpetersenii*	FMAS_AP4	85,96	88,75	329	33780	40.2	3.7
DDA 10944/10	*L. santarosai*	serovar Shermani str. LT 821	75,71	76,11	448	64782	42.2	3.8
LA 9525/17	*L. borgpetersenii*	FMAS_AP4	85,98	88,77	365	33924	40.2	3.8
PM 155/20	*L. borgpetersenii*	FMAS_AP4	85,96	88,75	316	35404	40.2	3.7

Table S8 NGS analysis results of Slovenian isolates from patients and reference *Leptospira* strains

Sample/ reference id	species	serovar	PubMLST MLST #1 (ST)	PubMLST MLST #2 (ST)	Pasteur Institute cgMLST (cgST)	loci matched[1]
SS 166/02	*L. interrogans*	copenhageni	17	47	*1002, 1398, 1400, 1529,* ***199****, 275, 749, 752, 755, 756, 757, 758, 759, 760, 777, 990, 997*	546[2]
ČP 226/02	*L. interrogans*	copenhageni	17	47	887	544/545 (99,8%)
OD 8720/11	*L. interrogans*	copenhageni	17	47	*1002, 1398, 1400, 1529,* ***199****, 275, 749, 752, 755, 756, 757, 758, 759, 760, 777, 990, 997*	546[2]
FF 291/02	*L. kirschnerii*		110	100	772	544/545 (99,8%)
SS 8049/14	*L. kirschnerii*		110	100	**771**+1[3]	543/545 (99,6%)
MJ 11721/08	*L. borgpetersenii*		155	181	783	536/545 (98,3%)
DDA 10944/10	*L. santarosai*		na	na	na	419/545 (76,9%)
LA 9525/17	*L. borgpetersenii*		155	181	783	536/545 (98,3%)
PM 155/20	*L. borgpetersenii*		155	181	783	536/545 (98,3%)

[1]Number of loci in isolate that matched the number of loci in cgMLST database of Institute Pasteur (% matched) (returned results after analysis at Institut Pasteur database)

[2] cgST compatible to isolate

[3] defined cgST plus one more comaptible to isolates´ genome

Table S9 Kraken2 taxonomic report

Percentage of fragments covered by the clade rooted at this taxon	Number of fragments covered by the clade rooted at this taxon	Number of fragments assigned directly to this taxon	Rank code	NCBI taxonomic ID number	Indented scientific name
5.68	620572	620572	U	0	unclassified
94.32	10300463	149	R	1	root
94.32	10300215	3928	R1	131567	cellular organisms
94.09	10275819	6290	D	2	Bacteria
90.61	9895322	0	P	203691	Spirochaetota
90.61	9895322	7	C	203692	Spirochaetia
90.61	9895140	0	O	1643688	Leptospirales
90.61	9895140	78	F	170	Leptospiraceae
90.61	9895062	91720	G	171	Leptospira
88.63	9679720	8221729	S	28183	Leptospira santarosai
13.35	1457991	0	S1	293071	Leptospira santarosai serovar Shermani
13.35	1457991	1457991	S2	758847	Leptospira santarosai serovar Shermani str. LT 821
0.49	53917	53917	S	28182	Leptospira noguchii
0.37	40284	37606	S	173	Leptospira interrogans
0.02	1946	1946	S1	312175	Leptospira interrogans serovar Bataviae
0.00	363	363	S1	338215	Leptospira interrogans serovar Bratislava
0.00	286	286	S1	214675	Leptospira interrogans serovar Manilae
0.00	35	23	S1	44275	Leptospira interrogans serovar Copenhageni
0.00	12	12	S2	267671	Leptospira interrogans serovar Copenhageni str. Fiocruz L1-130

0.00	25	2	S1	176	Leptospira interrogans serovar Hardjo
0.00	15	15	S2	38347	Leptospira interrogans serovar Hardjo-prajitno
0.00	8	8	S2	1279460	Leptospira interrogans serovar Hardjo str. Norma
0.00	10	10	S1	211880	Leptospira interrogans serovar Canicola
0.00	8	2	S1	57678	Leptospira interrogans serovar Lai
0.00	4	4	S2	189518	Leptospira interrogans serovar Lai str. 56601
0.00	2	2	S2	573825	Leptospira interrogans serovar Lai str. IPAV
0.00	5	0	S1	290341	Leptospira interrogans serovar Linhai
0.00	5	5	S2	1395589	Leptospira interrogans serovar Linhai str. 56609
0.10	11416	11416	S	28184	Leptospira weilii
0.09	9498	9497	S	1137606	Leptospira mayottensis
0.00	1	1	S1	1192864	Leptospira mayottensis 200901116
0.05	5771	5733	S	174	Leptospira borgpetersenii
0.00	32	32	S1	280504	Leptospira borgpetersenii serovar Javanica
0.00	6	6	S1	508536	Leptospira borgpetersenii serovar Ceylonica
0.01	1009	1009	S	29507	Leptospira kirschneri
0.01	862	862	S	408139	Leptospira kmetyi
0.01	754	754	S	2564040	Leptospira tipperaryensis
0.00	65	0	G1	2633828	unclassified Leptospira
0.00	53	53	S	1513297	Leptospira sp. GIMC2001

0.00	12	12	S	2838238	Leptospira sp. mild_001
0.00	41	41	S	1917830	Leptospira kobayashii
0.00	5	0	S	172	Leptospira biflexa
0.00	5	5	S1	145259	Leptospira biflexa serovar Patoc

Table S10 Reference sequences of *Leptospira* strains used for phylogenetic analysis.

Sample/ reference id	species	serovar
GCF_014858915.1	*L. interrogans*	Canicola
GCF_002073495.2	*L. interrogans*	copenhageni
GCF_000216355.1	*L. interrogans*	pomona
GCF_001995205.1	*L. interrogans*	australis
GCF_000244055.1	*L. interrogans*	autumnalis
GCF_000243515.1	*L. interrogans*	pyrogenes
GCF_014858935.1	*L. interrogans*	bataviae
GCF_014858895.1	*L. interrogans*	icterohaemorrhagiae
GCF_000244115.1	*L. interrogans*	hebdomadis
GCF_001010765.1	*L. interrogans*	bratislava
GCF_015162955.1	*L. borgpetersenii*	tarassovi
GCF_003046425.1	*L. borgpetersenii*	javanica
GCF_000244495.1	*L. borgpetersenii*	castellonis
GCF_000243695.2	*L. kirschnerii*	cynopteri
GCF_000243855.1	*L. kirschnerii*	grippotyphosa
GCF_000313175.2	*L. santarosai*	shermani
GCF_000306255.2	*L. noguchii*	panama
GCF_000017685.1	*L. biflexa*	patoc1
